# The moderation effect of entrepreneurial resilience on the relationship between financial literacy and sustainable performance

**DOI:** 10.3389/fpsyg.2022.954841

**Published:** 2022-08-15

**Authors:** Ali Saleh Alshebami, Majid Murad

**Affiliations:** ^1^The Saudi Investment Bank Chair for Investment Awareness Studies, The Deanship of Scientific Research, The Vice Presidency for Graduate Studies and Scientific Research, King Faisal University, Al-Ahsa, Saudi Arabia; ^2^Applied College in Abqaiq, King Faisal University, Al-Ahsa, Saudi Arabia; ^3^Department of Management Sciences, Muhammad Nawaz Sharif University of Engineering and Technology, Multan, Pakistan

**Keywords:** financial literacy, entrepreneurial resilience, sustainable performance, PLS-SEM, SME sectors, Saudi Arabia

## Abstract

The study intends to examine the role of financial literacy in sustainable performance of SME’s in Saudi Arabia, with the moderating effect of entrepreneurial resilience. The data for this study were gathered from 203 different SME’s sector entrepreneurs in Saudi Arabia using a convenience sampling technique. The hypothesis was tested through Smart-PLS software 3.3.9 version and structural equation modeling technique was used to verify the hypothesis relationships. The findings show that financial literacy has a significant and positive impact on sustainable performance. Moreover, results indicate that entrepreneurial resilience has a significant and positive effect on sustainable performance. Furthermore, the findings show that entrepreneurial resilience moderates the relationship between financial literacy and sustainable performance in Saudi Arabia. Lastly, this research article addressed the discussion and practical implications of the study.

## Introduction

Small and medium enterprises not only generate money, but also solve social and economic challenges associated to poverty (Fatoki, 2018). Many researchers argue that small and medium enterprises play a crucial role in stimulating economic development and create jobs at a cheap cost, distribute income evenly, and contribute to national wealth creation (Abor and Quartey, 2010; Ayala and Manzano, 2014; Ardito et al., 2018). Small and medium enterprises are well-positioned to service both local and regional markets because they utilize and add value to national resources (Corner et al., 2017; Fatoki, 2018). Recently, the economy of Kingdom of Saudi Arabia (KSA) has been increased due to the importance of small-and medium-sized businesses, which have a significant effect on the GDP in recent years. Investment in SMEs is on the rise in the sovereignty of Saudi Arabia. According to the latest plan, the contribution of SMEs to Saudi Arabia’s national economy is to increase from 20 to 35% and the unemployment rate is to be decreased from 11.6 to 7%. In Saudi Arabia, a large number of micro-entrepreneurs still face a number of obstacles with regard to entrepreneurial competences, access to financial literacy, entrepreneurial resilience, and sustainable performance of their businesses (Fletcher and Sarkar, 2013). Small and medium enterprises are not receiving loans from formal financial institutions for a variety of reasons: insufficient collateral, higher transaction costs, poor financial literacy, business records, and a lack of entrepreneurial resilience (Laghouag, 2022).

Typically, micro-entrepreneurs put tremendous effort into their operations, which they regard as an essential contributor to both local and national economic development. Due to their reliance and value addition to national resources, small businesses are ideally positioned to serve both local and regional needs (Fatoki, 2018). ﻿﻿﻿ SMEs play a vital role in the national economies of Saudi Arabia and cannot be overemphasized. According to Alsulami (2014), SMEs employ more than 4.5 million people, representing more than 80% of the total workforce, most of whom are foreign nationals. The management and bank, in order to foster the growth of informal businesses and SMEs, provide much-needed funding to these entities. Various programs of business assistance have been introduced, for instance, in the Kingdom of Saudi Arabia that aim at improving the performance of small and medium enterprises, fostering entrepreneurial skills, and providing training to entrepreneurs (Salem, 2014). Since the past few years, Saudi Arabia has pursued an aggressive policy of moving toward a knowledge-based economy. It is an important move by the government to assign various private and government institutions with the responsibility of guiding the framework of an economic perspective that aims to ensure economic growth and development (Segal and Gerstel, 2020).

Despite the fact that SMEs contribute substantially, research indicates that they face many obstacles and hurdles as a consequence of the COVID-19 pandemic (Adam and Alarifi, 2021). Lockdowns and movement limitations imposed by governments in many countries have caused small- and medium-sized enterprises (SMEs) to cease operations, diminished their financial stability, and increased their financial risk (Omar et al., 2020). A labor and input shortage has impacted SMEs due to supply chain disruptions, adversely affecting their sales and their ability to convene their financial responsibilities and pay their workers. In Saudi Arabia as well, small- and medium-sized companies have experienced problems regarding financial performance. Business conditions in Saudi Arabia are very hard and volatile, and many small- and medium-sized enterprises (SMEs) face hostile conditions rather than favorable conditions. SMEs are incapable of creating long-term employment and eradicating poverty due to their high failure rate and poor performance. It is common for entrepreneurs to encounter high levels of stress, a variety of barriers, and a great deal of uncertainty about their projected outcomes. It is important to understand how entrepreneurs cope with unpredictability and what inspires them.

Often, entrepreneurs make mistakes and misjudge the outcome of their decisions due to ambiguous or incomplete information available to them. To remain competitive, entrepreneurs need to continuously adjust their goals and strategies (Alharbi et al., 2018). In order to succeed as an entrepreneur, one should have a high level of entrepreneurial resilience, which is distinct as the capacity to rapidly respond to and overcome failures. Entrepreneurial resilience is believed to be an essential component of success (Savlovschi and Robu, 2011). In contrast, past empirical research on the association between entrepreneurial resilience and corporate performance has produced contradictory findings (Ayala and Manzano, 2014). To be successful as an entrepreneur, one must not only achieve professional milestones, but also personal ones. It is not adequate to center of attention exclusively on the organizational level. It is critical to integrate both micro and macro perspective indicators for a comprehensive understanding of the interaction between the entrepreneur and their enterprise.

According to Fisher et al. (2016), resilience is significantly associated with business success. A successful and long-term business venture necessitates the presence of individuals with distinct talents and characteristics, some of which have been shown to be extremely important in achieving various levels of organizational success (Beattie, 2016). This may be why the topic of micro-enterprise success has grown in popularity among entrepreneurs and researchers, particularly those looking for a definitive formula for success in micro-entrepreneurship (Webb et al., 2013). Sustainable firms must have knowledge and skills regarding entrepreneurial competencies and competitive strategies in order to perform well (Burnard and Bhamra, 2011). However, informal business owners and managers in the Kingdom of Saudi Arabia frequently lack the necessary skills and strategies (Alshebami, 2022). However, despite the necessity of entrepreneurial skills, a lack of knowledge renders some entrepreneurs incapable of creativity, unable to take risks, unmotivated, and hesitant to acquire new skills (Naffziger, 1995). Entrepreneurs must be vigilant, innovative, imaginative, and diligent, as well as have the mental capability of detecting, evaluating, and maximizing opportunities and converting them into competitive and efficient strategies as well as profitable performance (Al Mamun et al., 2018).

In emerging economies, such as the Kingdom of Saudi Arabia, financial literacy and entrepreneurial skills are essential to the success of small businesses and to improve their economic performance. Research indicates that informal firms in Saudi Arabia receive less attention in regard to how informal business influences entrepreneurial behavior, such as entrepreneurial resilience and access to finance, on the sustainable performance of small companies. The phenomenon of enterprising small firms driving economic growth has received relatively little attention in the Arab world, particularly in Saudi Arabia. Few studies have examined the effect of entrepreneurial resilience, and the sustainability of small businesses (Hayward et al., 2010; Fatoki, 2018). To boost the economic performance of entrepreneurship, it is crucial to bridge this gap, given that small businesses need a variety of skills, entrepreneurial resilience, and financial literacy. In this study, we propose three objectives. First, we examined the direct influence of financial literacy on small business sustainability performance. Second, we examined the direct effect of entrepreneurial resilience on sustainable performance. Third, we also aimed to assess whether entrepreneurial resilience plays a moderating role in the relationship between financial literacy and sustainable performance of SMEs in Saudi Arabia.

## Literature review and hypotheses development

### Financial literacy and sustainable performance

Financial literacy can play a significant role in determining access to finance (Abdullah and Chong, 2014). Low financial literacy may stymie the adoption of more complex monetary products, such as health coverage, because consumers may be cautious to purchase a product whose utility they do not fully comprehend. According to De Mel et al. (2008), financial literacy is a critical component of SME growth and an important indicator of productivity. Financial literacy, popularly understood as the capacity to obtain, understand, and assess the meaningful information necessary for making sound financial decisions and choices with an understanding of their probable financial implications, is vital for the growth of SMEs in emerging countries (Hsu, 2016).

The importance of financial management skills has increased in recent years for small business owners in developing nations (Bruhn and Zia, 2013; McKenzie and Woodruff, 2014). The aim of financial literacy is to provide managers with the necessary information so that they can create budgets, save money, and make wise investment decisions (Adomako et al., 2016). Financial literacy also increases the effectiveness and quality of a enterprise’s monetary processes, as well as the accuracy of its objective reporting (Adomako et al., 2016). As per Siekei et al. (2013), financial literacy assists SMEs in preparing for difficult financial times by providing risk-mitigation strategies such as saving, diversifying assets, and avoiding over-indebtedness. According to Oseifuah (2010), entrepreneurs of all ages make decisions about resource acquisition, allocation, and utilization on a regular basis. Entrepreneurs must be financially literate because their activities always have financial consequences.

Bongomin et al. (2017) argue that financial literacy improves decision-making processes such as timely bill payment and effective debt strategic planning, which increases the credit worthiness of SMEs to support livelihoods, good financial systems, and poverty reduction. According to Siekei et al. (2013), financial literacy program management skills can help SME owners maintain their loan portfolios in such a way that loan obligations and interest expenses are minimized. A financial literacy program is critical for the development of knowledge and skills needed by SMEs to effectively plan for the future, establish a savings plan, and make strategic investment decisions (Greenspan, 2002). Moreover, it is claimed that people who are financially literate make better financial decisions and make fewer management mistakes than those who are not (Njoroge and Gathungu, 2013). Furthermore, financial literacy facilitates the development of risk management skills among SMEs, which has a significant role in the sustainable performance, therefore we hypothesize that:

*H1*: Financial Literacy has significant and positive effect on sustainable performance.

### Entrepreneurial resilience and sustainable performance

The word resilience comes from the Latin verb “resilire,” which meaning “to bounce back” (Bullough and Renko, 2013; Nchabeleng et al., 2018). Tonis (2015) suggests that the idea of resilience is drawn from material physics, residual stress, or the capacity of materials to tolerate and absorb stress, which is referred to as “resilience.” The notion of resilience was presented to the psychology community, Fletcher and Sarkar (2013) introduced the concept of resilience to the psychology community. In psychology, resilience is the ability to deal with trauma and stress in a healthy way. In other words, resilience is a coping mechanism for dealing with change, hardship, and opportunity. It has to do with a person’s capacity to move on from a difficult situation (Windle, 2011). Although the concept can be operationalized in a variety of ways, the majority of formulations are based on two basic concepts: adversity and positive adaptation. This is the ability to bounce back from difficult situations, deal with adverse situations, and achieve positive outcomes in the context of resilience. The capacity to overcome adversity and achieve success is defined as resilience in this study. This multidisciplinary concept of resilience is still being fleshed out (Tedeschi and Calhoun, 2004).

Resilience theories often combine psychological concepts and overlap with other scientific fields. There are several resilience theories and models in the literature (Richardson, 2002; Branicki et al., 2017). The resilience model’s family adjustment and adaption response and the grounded theory of human resilience is one example among many others. The majority of resilience theories are group-specific. People and events are different, thus we need a universally applicable theory of resilience (Abebrese, 2015). Moreover, Abebrese (2015) argues that a person’s ability to cope with stress begins with having a comfort zone in which they are mentally, physically, and spiritually in balance. After a period of time, individuals who have experienced disruption should be able to adjust and reintegrate. This process will result in one of four outcomes. First, we have resilient integration, which is the emergence of additional protective mechanisms in response to disturbances. Second, “homeostasis reintegration” refers to people staying in their familiar environments following a shock or shocks to their routines. Thirdly, there is loss of reintegration. The protecting elements of the system were destroyed during the disruption phase. Fourth, dysfunctional reintegration occurs when individuals resume destructive behaviors following a disturbance (Richardson, 2002; Fatoki, 2018). Therefore, the following hypothesis is predicted:

*H2*: Entrepreneurial resilience has a positive and significant effect on sustainable performance.

### Moderating role of entrepreneurial resilience

Entrepreneurial resilience refers to a set of dynamic adaptive mechanisms that enable entrepreneurs to remain forward-looking when confronted with unfavorable economic conditions and market disruptions (Bernard and Barbosa, 2016). A key element of entrepreneurial resilience is the ability to deal with unpleasant personal, business, and disaster circumstances while remaining optimistic. An entrepreneur who is resilient embraces changes rather than resists it. Resilient entrepreneurs endeavor to accomplish their objectives and conquer barriers. Further, resilient entrepreneurs often have a high stage of patience for uncertainty (Denz-Penhey and Murdoch, 2008). Entrepreneurs who are resilient develop a positive mentality rather than acting out of fear, indifference, or desperation. In an uncertain and rapidly changing business environment, resilient entrepreneurs are well prepared (Morisse and Ingram, 2016). The characteristics of resourcefulness and optimism are important to entrepreneurial resilience.

“Hardiness” refers to an entrepreneur’s capacity to exercise personal control over his or her business without relying on outside help. Entrepreneurs who are resourceful are well-equipped to deal with a wide range of problems. Entrepreneurial resilience is characterized as the capacity to deal with change and uncertainty in the business climate; (2) the requirement to sustain one’s physical and mental health despite frequent business pressure and expectations; (3) the ability to recover from individual and commercial setbacks; and (4) the ability to shift to a new way of managing the business if the previous method failed to meet the specific goals. Based on the characteristics of entrepreneurs’ personalities and the attribution theory, several studies have proposed a relationship between entrepreneurial resilience and a psychological attribute (Ofunoye, 2017). Trait theory suggests that successful entrepreneurs possess a set of characteristics or traits that contribute to their success. These include the ability to take risks, the ability to establish and manage an organization effectively, perseverance and resilience, as well as the ability to see the future (Davidsson, 2005; Buang, 2012; Heider, 2013). Thus, this study proposed the following hypothesis:

*H3*: Entrepreneurial resilience will moderate the relationship between financial literacy and sustainable performance.

### Conceptual model

**Figure 1:**
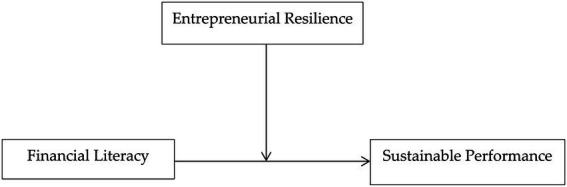
Conceptual model. Authors’ elaboration.

Figure 1 depicts the conceptual model used to investigate the impact of financial literacy on sustainable performance with the moderating influence of entrepreneurial resilience of small businesses in Saudi Arabia.

## Research methodology

### Sample and data collection

This study was quantitative in nature and used cross-sectional data. An online questionnaire survey was conducted from April 1, 2022, to May 30, 2022, using a non-probability convenience sampling technique. The survey’s original draft was written in English, and it was translated using a translation and back translation process by different language experts with strong Arabic and English translation skills. Hence, the final sample size included 203 valid responses of entrepreneurs. The participation of the entrepreneurs was voluntary and we assured them these data were purely used for academic purposes.

Among the valid responses, 70.9% were male and 29.1% were female. In terms of business sectors such as retail and wholesalers (13.3%), services (13.3%), construction (10.8%), production (16.7%), insurance and finance (4.9%), and other sectors (40.9%). The highest rate of year established was between 6 and 10 years (45.3%) and lowest rate of year established was between less than 5 years (39.9%). 26.6% had secondary school education, 10.3% had higher school education, 22.2% had diploma degree, and 40.9% had bachelor degree or above. Lastly, in terms of age groups were less than 5 years (2.5%), 21–30 years (53.7%), 31–40 years (34%), 41 to 50 years (8.9%), and 51–60 above years (1.0%).

### Measures

The items measuring the constructs were adapted from existing measures except exposure condition which was drawn from the literature with adjustments to fit the study context. All measurement items were anchored on a 5-point Likert “strongly disagree to strongly agree” scale (Appendix). Control variables such as age, gender, marital status, business sectors, education, and firm established were not of direct theoretical interest and analyzed in structural model analysis.

#### Entrepreneurial resilience

To assess entrepreneurial resilience, we used nine measurement constructs from the existing study by Fatoki (2018). A sample item “I can handle unpleasant feelings.” The Cronbach’s alpha for entrepreneurial resilience was 0.923.

#### Financial literacy

We used a seven-item scale from a study to assess financial literacy (Chia et al., 2011). A sample item “I have better understanding of how to invest my money.” The Cronbach’s alpha for financial literacy was 0.914.

#### Sustainable performance

We used a five-item scale to assess sustainable performance, adapted from a study by (Morgan and Strong, 2003). A sample item “compared to major competitors my firm possesses a relatively higher level of environmental performance.” The Cronbach’s alpha for sustainable performance was 0.912.

### Common method bias

As the study data are based on self-reports, in order to ensure the sufficiency of the scale reliability and convergent validity as well as instill confidence in the study findings, preventive measures procedural and statistical were taken to lessen possible method variance. To do this, during data collection, the scale items were randomly mixed in the survey. Also, we checked for the presence of common method variance during the data analyses. The result of a Harman’s single-factor test was that no single factor emerged after entering all of the key variables into an exploratory factor analysis (EFA). Furthermore, the “forced” single-factor solution only accounted for 33.23% of variance, well below the recommended 50% threshold (Podsakoff et al., 2003).

## Results

### Data analysis method

The study’s hypotheses were tested using Smart PLS version 3.3.9, including confirmatory factor analysis, hypothesis checking, validity estimation, moderation testing, and internal accuracy. According to previous researchers, PLS avoids many of the restrictive assumptions that underpin maximum likelihood methods and protects against inaccurate solutions and factor indeterminacy (Hair et al., 2014; Sarstedt et al., 2014). PLS-SEM does not make any distributional assumptions about the error terms, and it can handle both reflecting and formative constructs (Henseler et al., 2016). Furthermore, unlike covariance-based SEM techniques, PLS is unaffected by sample size constraints and can be used with samples larger than thirty. Our sample size is 203 people, so we have a sample that requires PLS-SEM (Sarstedt et al., 2014).

### Measurement model

**Figure 2:**
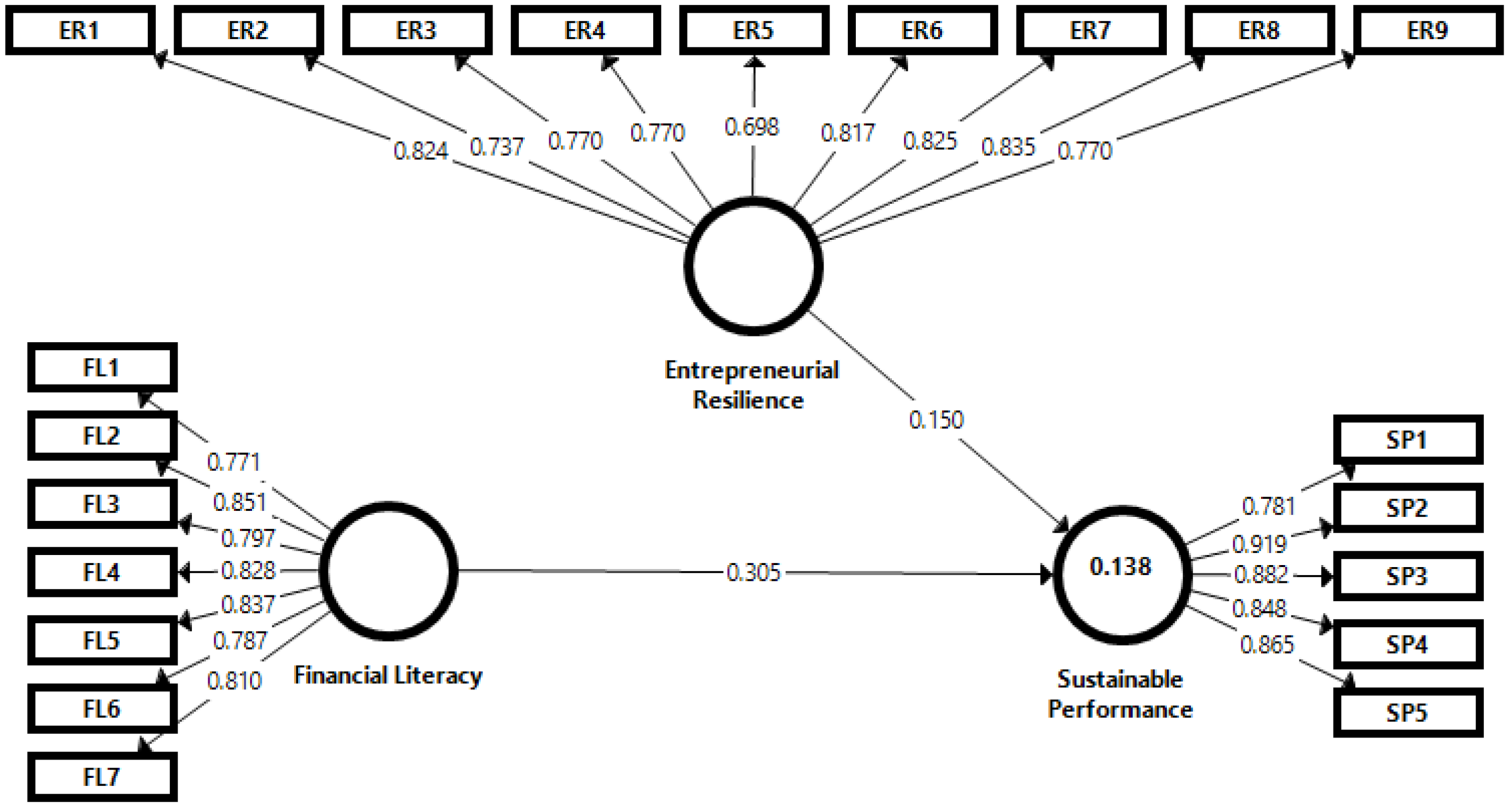
Structural model. Authors’ elaboration.

To assess reliability, the composite scale reliability (CR), Cronbach’s alpha (CA), and average variance extracted (AVE) were used. Table 1 and Figure 2 show that all constructs CR were greater than the threshold value of 0.70, Cronbach’s alpha CA was greater than the threshold value of 0.70, and AVE was greater than the threshold value of 0.50 (Hair et al., 2014; Henseler et al., 2016). Furthermore, we evaluated convergent validity by examining the standardized loadings of the measures on their respective constructs, and we discovered that they all exceed 0.70 (Sarstedt et al., 2014).

**Table 1:** Measurement model.

Constructs	Loadings	Cronbach’s alpha	Composite reliability	Average variance extracted (AVE)
Entrepreneurial resilience		0.923	0.935	0.615
	ER1	0.824		
	ER2	0.737		
	ER3	0.770		
	ER4	0.770		
	ER5	0.698		
	ER6	0.817		
	ER7	0.825		
	ER8	0.835		
	ER9	0.770		
Financial literacy		0.914	0.931	0.659
	FL1	0.771		
	FL2	0.851		
	FL3	0.797		
	FL4	0.828		
	FL5	0.837		
	FL6	0.787		
	FL7	0.810		
Sustainable performance		0.912	0.934	0.740
	SP1	0.781		
	SP2	0.919		
	SP3	0.882		
	SP4	0.848		
	SP5	0.865		

Primary data.

Moreover, Table 2 presents the correlation between all three constructs, demonstrating discriminant validity. AVE for each dimension should be predicted to be higher than the squared correlation between components to completely meet the standards for discriminant validity (Fornell and Larcker, 1981; Hair et al., 2014). None of the constructs’ inter-correlations exceeded the square root of the model’s constructs’ AVE. Furthermore, we examined the standardized loadings of the measures on their respective constructs to determine convergent validity, and we revealed that all of the indicators have standardized factor loading greater than 0.70.

**Table 2:** Discriminant validity (Fornell–Larcker Criteria).

	Entrepreneurial resilience	Financial literacy	Sustainable performance
Entrepreneurial Resilience	0.784		
Financial Literacy	0.243	0.812	
Sustainable Performance	0.224	0.342	0.860

Primary data.

### Structural model

Various quality scores, such as the coefficient of determination (*R*^2^), predictive validity (*Q*^2^), and SRMR are used to verify the structural model results. The values of *R*^2^ and *Q*^2^ are shown in Table 3. The endogenous constructs R^2^ values were used to assess model fit and determine how well data points match a line or curve. According to Chin (1998), *R*^2^ levels can be classified as small (0.02 < *R*^2^ < 0.13), medium (0.13 < *R*^2^ < 0.26), or large (0.26 < *R*^2^). The endogenous constructs’ *R*^2^ statistic values were utilized to test model fit (Chin, 1998). The value of sustainable performance (*R*^2^ = 0.138) indicates medium effect size. Moreover, the dependent construct *Q*^2^ validity was similarly satisfactory. This result indicates that *Q*^2^ result was also satisfactory and explains (0.112) variation in the dependent variable. Moreover, SRMR (standardized root mean squared residual) should be equal to or less than 0.08 (Henseler et al., 2016), and results indicate that SRMR for our model is 0.063, which meets this criterion.

**Table 3:** Structural model.

Hypotheses	(*R*^2^ = 0.138 and *Q*^2^ = 0.112)	*β*	*t*	*p*
	*Direct relationships*			
H1	Financial Literacy → Sustainable Performance	0.338	5.222	0.000
H2	Entrepreneurial Resilience → Sustainable Performance	0.180	1.965	0.049
	*Moderating relationships*			
H3	Entrepreneurial Resilience Financial Literacy → Sustainable Performance	0.113	1.088	0.042

Primary data.

### Hypothesis testing

**Figure 3:**
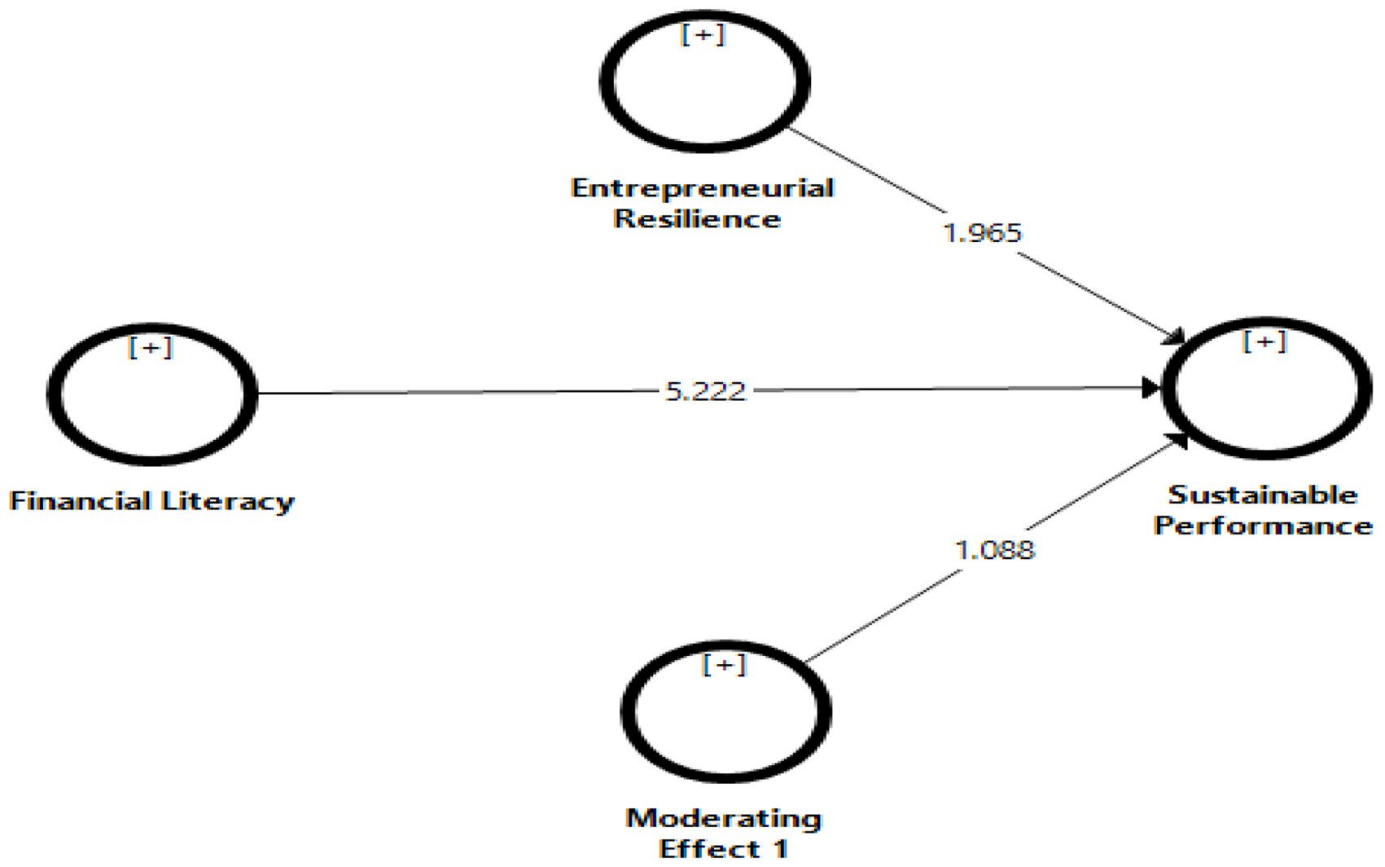
Bootstrapping. Authors’ elaboration.

PLS-SEM partial least squares structural equation modeling was used to test the hypothesis relationships using the 5,000 bootstrapping method. Table 3 and Figure 3 display the results. The findings were all statistically significant. Financial literacy had a positive and significant impact on long-term performance (*β* = 0.338; *t* = 5.222; *p* = 0.000), according to the results of the H1 test. As a result, H1 was approved. Meanwhile, findings from H2 reveal that entrepreneurial resilience has a positive and significant impact on long-term performance (*β* = 0.180; *t* = 1.965; *p* = 0.049). As a result, H2 was approved. Furthermore, H3 results reveal that entrepreneurial resilience positively moderates the relationship between financial literacy and long-term performance (*β* = 0.113; *t* = 1.088; *p* = 0.042). Thus, H3 was also supported.

## Discussion

Along with other skills, the level of financial literacy of the entrepreneur is a crucial factor in ensuring the success of the firm. People can acquire the capability of financial literacy over time (Elert et al., 2015; Anderson et al., 2018). According to findings, financial literacy has a significant impact on firm growth. Individuals with little financial experience are more likely to make poor monetary decision-making. Individuals with (above average) financial literacy, on the other hand, have the opportunity to closely examine the risk and make timely decisions about the firm’s value (Joseph, 2017). The hypothesis was validated and the study’s objective was met after analyzing the effect of financial literacy on enterprise success.

The findings, however, are consistent with previous research on the influence of financial literacy on business performance (Van Rooij et al., 2011; Njoroge and Kagiri, 2017; Sajuyigbe et al., 2020). According to the findings of this study, financial literacy appears to have a significant impact on sustainable performance. As a result, the findings are consistent with previous research and literature, indicating that financial literacy contributes to business success (Sabana, 2014; Eniola and Entebang, 2016). Financial literacy has a statistical significant impact on the outcome of entrepreneurial businesses, according to existing research.

Moreover, this study found to gain an understanding that how financial literacy influences entrepreneurial resilience and sustainable performance. Our research shows that entrepreneurial resilience moderates the link between financial literacy and sustainable performance in such a way that the association becomes stronger and more significant. Moreover, it revealed a positive and significant moderating effect of entrepreneurial resilience on financial literacy and long-term performance. Our results suggest that entrepreneurial resilience contributes to a firm’s growth by enhancing the entrepreneurs’ financial literacy (Hilgert et al., 2003). As a result, financial literacy is likely to reduce the need for risk tolerance, allowing entrepreneurs to make important decisions that benefit the firm’s growth. This study supports the findings (Christelis et al., 2010; Van Rooij et al., 2011) that entrepreneurial resilience moderates the effect of financial literacy and firm growth, shedding light on the significance of financial literacy in decision-making and behavior (Cole et al., 2009; Drexler et al., 2014).

As a result, entrepreneurship theory is unlikely to produce novel insights into the role of financial literacy in connectivity to the finance-growth relationship in developing countries. Furthermore, entrepreneurial resilience significantly moderated the relationship between financial literacy and long-term performance. Financial resources are required for both the acquisition of resources and capabilities, as well as the coordination of other resources (Brinckmann et al., 2011). Financial literacy improves entrepreneurial resilience during decision-making (Rahmandoust et al., 2011). A thorough understanding of finance allows you to assess the risk scenario and helps entrepreneurs control their emotions and practice locus of control during an understanding risk in order to make a critical choice to avoid financial loss. It is critical to use the mental model to determine whether an entrepreneur has poor financial literacy so that you can help, support, or improve their decision-making processes. Eniola and Entebang (2016) argued that financial literacy is necessary. Since these individuals are more knowledgeable of financial topics, they participate in the financial markets more frequently. Lusardi and Mitchell (2007) examined how borrowers’ levels of financial literacy and high costs affected their decision to borrow. It is crucial for the financial success of small- and medium-sized enterprises (SMEs), as well as a tool for combating poverty in developing economies, to possess financial literacy. Financial literacy relies on the ability to use knowledge and skills to manage financial resources effectively. Current research concludes that SMEs led by entrepreneurs who are financially literate have a greater chance of achieving success than those who are illiterate.

Entrepreneurial resilience enables businesses to achieve sustainable performance. In this study, the findings support the importance of entrepreneurial resilience on the sustainability of the performance of SMEs in the examined context. It is evident from these findings that entrepreneurial resilience positively affects SMEs’ success (Fatoki, 2018; Borbolla-Albores and Reyes-Mercado, 2022). Small- and medium-sized enterprises perform better when entrepreneurial resilience is present. As entrepreneurs with resilience, they apply criteria regarding how to develop excellent connections in society, emphasizing societal ideals over personal interests, while still seeking to achieve organizational success by utilizing available opportunities. Increasing resilience enhances creativity and decision-making abilities. Our findings indicate that persistent entrepreneurial behavior is the result of early life experiences that contribute to the development of entrepreneurial resilience (Fisher et al., 2014).

Additionally, the results are identical to those reported in which states entrepreneurial resilience is a predictor of organizational success. SME owners with a strong entrepreneurial spirit are better positioned to lead their companies to success (Morisse and Ingram, 2016). Entrepreneurs who manage SMEs are successful because of their ability to adapt quickly to change, deal with stress and personal emotions, recover quickly from failure, and also have a great sense of humor. Furthermore, small businesses are prone to growth because of the openness of their owners. Further, the results are comparable with those reported in (Bullough and Renko, 2013), which point out a positive relationship between entrepreneurial resilience and individual achievement. The entrepreneurial resilience of SMEs may encourage their employees to work together to achieve organizational objectives.

## Implications

### Theoretical implications

Theoretically, the contribution of this study is in the form of empirical evidence that ratifies and extends the adoption of the trait theory in investigating the effect of financial literacy on sustainable performance among small and medium enterprises in Saudi Arabia. Based on the extensive review of literature, this appears to be the first study that employed primary data to identify and to provide empirical support for correlations between financial literacy, entrepreneurial resilience, and sustainable performance, within the context of emerging economies, using small and medium enterprises in Saudi Arabia as its data source. From the conceptual perspective, this study contributes crucially toward the trait theory from the stance of sustainable performance. This is performed by theorizing and providing empirical evidence that successful entrepreneurs possess a set of characteristics that contribute to their success. Therefore, this study fulfills the ultimate purpose of the theory toward attaining sustainable performance and competitive advantage. In terms of novelty, this study uniquely contributes toward the body of knowledge by evaluating the direct effect of financial literacy and moderating role of entrepreneurial resilience on sustainable performance of small and medium enterprises in Saudi Arabia.

### Practical implications

In terms of practical implications, the study is expected to broaden our understanding of the various factors that affect the success and sustainability of micro-enterprises. In recent years, the KSA government has become increasingly involved in promoting small businesses. There is a strong link between financial literacy and economic development, according to the findings of this study. International financial institutions such as the World Bank have emphasized this perspective both conceptually and practically. The study has some implications as a result of its findings. From a practitioner’s perspective, the findings of this study suggest that financial literacy can help businesses implement effective financial management by strengthening the link between access to capital and enterprise growth. Entrepreneurs of small- and medium-sized enterprises (SMEs) need this understanding to acquire, study, and apply new financial information to improve the efficiency and quality of their financial services in order to promote development. Second, entrepreneurs with strong financial management practices can better analyze and compare financial products like bank accounts, savings accounts, credit and loan product lines, payment instruments, investment opportunities, and insurance coverage. The Kingdom of Saudi Arabia and other developing countries may want to focus more on both capital availability and financial literacy among entrepreneurs and policymakers. According to the findings of this study, entrepreneurs with financial literacy and access to capital have high business performance. SME entrepreneurs can also improve their financial knowledge and learn how to raise capital.

According to the study, entrepreneurs who rely solely on financial capital and ignore the importance of financial literacy are unlikely to succeed. As a result, while entrepreneurs have easy access to financial resources, they should also have entrepreneurial skills and financial literacy to help their businesses grow. Finally, the study’s findings have implications for entrepreneurs looking to impress venture capitalists so that they will be more likely to invest in their enterprise. In order for venture capitalists to determine the feasibility of entrepreneurs’ business models, they evaluate their business models, competencies, and financial forecasts. To accomplish this goal, the entrepreneur must be resilient and have solid financing capabilities to persuade venture capitalists to invest. These findings are expected to impact venture capitalists in developing countries in general, as well as the Kingdom of Saudi Arabia in particular. This data may be used by policymakers to address the economic difficulties faced by Saudi Arabian entrepreneurs with low incomes. To encourage low-income families to participate in entrepreneurial endeavors, underlying organizations should provide training, financial education, and legislation to help develop entrepreneurial skills and resilience.

## Conclusion, limitations, and future research directions

There are some limitations to this study that should be taken into account in future research directions. First, this study used cross-sectional data from Saudi Arabia to verify the significance of the relationship between the variables under consideration; future research could take a longitudinal approach to investigate the impact of financial literacy on sustainable performance in various countries. Second, using a small sample size, we only looked at the moderating role of entrepreneurial resilience in determining the relationship between financial literacy and sustainable performance in Saudi Arabia. Future researchers can contribute by additional entrepreneurial traits such as entrepreneurial mindset, entrepreneurial orientation that directly or indirectly impact their sustainable development. Third, this study adopts a quantitative approach to analyze the primary data; future research can encompass both qualitative and quantitative to extend the understanding of several contextual components related to sustainable performance. Future research can further explore the topic among multi-samples or comparative-based analysis among the provinces or countries to add more contribution in the field of entrepreneurship and sustainable growth.

## Data availability statement

The raw data supporting the conclusions of this article will be made available by the authors, without undue reservation.

## Ethics statement

The studies involving human participants were reviewed and approved by Ethics Committee of the King Faisal University, Saudi Arabia. Written informed consent to participate in this study was provided by the participants.

## Author contributions

AA proposed the idea of research model and wrote the manuscript. MM designed and carried out the methodology and results and extensively edited the manuscript. All authors contributed to the article and approved the submitted version.

## Funding

This work was supported by the Saudi Investment Bank Chair for Investment Awareness Studies, the Deanship of Scientific Research, and the Vice Presidency for Graduate Studies and Scientific Research, King Faisal University, Saudi Arabia (Grant No. 35).

## Conflict of interest

The authors declare that the research was conducted in the absence of any commercial or financial relationships that could be construed as a potential conflict of interest.

## Publisher’s note

All claims expressed in this article are solely those of the authors and do not necessarily represent those of their affiliated organizations, or those of the publisher, the editors and the reviewers. Any product that may be evaluated in this article, or claim that may be made by its manufacturer, is not guaranteed or endorsed by the publisher.

## Appendix

**Table:**

Constructs	Measures	1	2	3	4	5
Entrepreneurial resilience						
ER 1	I can achieve goals despite obstacles.					
ER 2	I am not easily discouraged by failure.					
ER 3	I think of myself as a strong person.					
ER 4	I can stay focused under pressure.					
ER 5	I tend to bounce back after illness or hardship.					
ER 6	I can deal with whatever comes my way.					
ER 7	I am able to adapt to change.					
ER 8	I try to see the humorous side of things.					
ER 9	I can handle unpleasant feelings.					
Financial literacy						
FL 1	I have better understanding of how to invest my money.					
FL 2	I have better understanding of how to manage my credit use.					
FL 3	I have a very clear idea of my financial needs during retirement.					
FL 4	I have the ability to maintain financial records for my income and expenditure.					
FL 5	I have little or no difficulty in managing my Money.					
FL 6	I have better understanding of financial instruments (e.g., bonds, stock, T-bill, future contract, option, etc.)					
FL 7	I have the ability to prepare my own weekly /monthly budget.					
Sustainable performance						
SP 1	Compared to major competitors my firm possesses a relatively higher level of environmental performance.					
SP 2	Compared to major competitors my firm possesses a relatively higher level of social performance.					
SP 3	Compared to major competitors my firm possesses a relatively higher level of retention of employees.					
SP 4	Compared to major competitors my firm possesses a relatively higher level of investment in society.					
SP 5	Compared to major competitors my firm possesses a relatively higher level of balance between financial, social, and environmental aspects.					
